# Broad-Spectrum Antiviral Therapeutics

**DOI:** 10.1371/journal.pone.0022572

**Published:** 2011-07-27

**Authors:** Todd H. Rider, Christina E. Zook, Tara L. Boettcher, Scott T. Wick, Jennifer S. Pancoast, Benjamin D. Zusman

**Affiliations:** Lincoln Laboratory, Massachusetts Institute of Technology, Lexington, Massachusetts, United States of America; Center for Disease Control and Prevention, United States of America

## Abstract

Currently there are relatively few antiviral therapeutics, and most which do exist are highly pathogen-specific or have other disadvantages. We have developed a new broad-spectrum antiviral approach, dubbed Double-stranded RNA (dsRNA) Activated Caspase Oligomerizer (DRACO) that selectively induces apoptosis in cells containing viral dsRNA, rapidly killing infected cells without harming uninfected cells. We have created DRACOs and shown that they are nontoxic in 11 mammalian cell types and effective against 15 different viruses, including dengue flavivirus, Amapari and Tacaribe arenaviruses, Guama bunyavirus, and H1N1 influenza. We have also demonstrated that DRACOs can rescue mice challenged with H1N1 influenza. DRACOs have the potential to be effective therapeutics or prophylactics for numerous clinical and priority viruses, due to the broad-spectrum sensitivity of the dsRNA detection domain, the potent activity of the apoptosis induction domain, and the novel direct linkage between the two which viruses have never encountered.

## Introduction

A serious threat is posed by viral pathogens, including clinical viruses (HIV, hepatitis viruses, etc.), natural emerging viruses (avian and swine influenza strains, SARS, etc.), and viruses relevant to potential bioterrorism (Ebola, smallpox, etc.). Unfortunately, there are relatively few prophylactics or therapeutics for these viruses, and most which do exist can be divided into three broad categories [1]–[3]: (1) Specific inhibitors of a virus-associated target (e.g., HIV protease inhibitors, RNAi) generally must be developed for each virus or viral strain, are prone to resistance if a virus mutates the drug target, are not immediately available for emerging or engineered viral threats, and can have unforeseen adverse effects. (2) Vaccines also require a new vaccine to be developed for each virus or viral strain, must be administered before or in some cases soon after exposure to be effective, are not immediately available for emerging or engineered viral threats, can have unforeseen adverse effects, and are difficult to produce for certain pathogens (e.g., HIV). (3) Interferons and other pro- or anti-inflammatories are less virus-specific, but still are only useful against certain viruses, and they can have serious adverse effects through their interactions with the immune and endocrine systems.

To overcome these shortcomings of existing approaches, we have developed and demonstrated a novel antiviral approach that is effective against a very broad spectrum of viruses, nontoxic *in vitro* and *in vivo*, and potentially suitable for either prophylactic or therapeutic administration. Our approach, which we call a **D**ouble-stranded **R**NA (dsRNA) **A**ctivated **C**aspase **O**ligomerizer (**DRACO**), is designed to selectively and rapidly kill virus-infected cells while not harming uninfected cells.

Our DRACO approach combines two natural cellular processes. The first process involves dsRNA detection in the interferon pathway. Most viruses have double- or single-stranded RNA (ssRNA) genomes and produce long dsRNA helices during transcription and replication; the remainder of viruses have DNA genomes and typically produce long dsRNA via symmetrical transcription [4]–[5]. In contrast, uninfected mammalian cells generally do not produce long dsRNA (greater than ∼21–23 base pairs) [4]–[5]. Natural cellular defenses exploit this difference in order to detect and to attempt to counter viral infections [6]–[7]. For example, protein kinase R (PKR) contains an N-terminal domain with two dsRNA binding motifs (dsRBM 1 and 2) and a C-terminal kinase domain [8]–[9]. Binding of multiple PKR proteins to dsRNA with a length of at least 30–50 base pairs [5] activates the PKRs via trans-autophosphorylation; activated PKR then phosphorylates eIF-2α, thereby inhibiting translation of viral (and cellular) proteins. Other examples of proteins that detect viral dsRNA include 2′,5′-oligoadenylate (2–5A) synthetases [10], RNase L (activated via dimerization by 2–5A produced by 2–5A synthetases in response to dsRNA [11]), TLR 3 [12], interferon-inducible ADAR1 [13], and RIG-I and Mda-5 [6]–[7].

The second natural process used by our approach is one of the last steps in the apoptosis pathway [14], in which complexes containing intracellular apoptosis signaling molecules, such as apoptotic protease activating factor 1 (Apaf-1) [15]–[16] or FLICE-activated death domain (FADD) [17]–[18], simultaneously bind multiple procaspases. The procaspases transactivate via cleavage, activate additional caspases in the cascade, and cleave a variety of cellular proteins [14], thereby killing the cell.

Many viruses attempt to counter these defenses. A wide variety of viruses target dsRNA-induced signaling proteins, including IPS-1, interferon response factors (IRFs), interferons and interferon receptors, JAK/STAT proteins, and eIF-2α [19]–[20]. Some viral products attempt to sequester dsRNA (e.g., poxvirus E3L [21]) or to directly interfere with cellular dsRNA binding domains (e.g., HIV TAR RNA [19]–[20]). Virtually all viruses that inhibit apoptosis do so by targeting early steps in the pathway, for example by inhibiting p53, mimicking anti-apoptotic Bcl-2, or interfering with death receptor signaling [22]–[23]. Among the few viral proteins that directly inhibit one or more caspases are African swine fever virus A224L (which inhibits caspase 3) [24], poxvirus CrmA (which inhibits caspases 1, 8, and 10 but not others) [25], and baculovirus p35 (which inhibits several caspases but is relatively ineffective against caspase 9) [25].

Because PKR activation and caspase activation function in similar ways and involve proteins that have separate domains with well-defined functions, these two processes can be combined to circumvent most viral blockades [26]–[27]. In its simplest form, a DRACO is a chimeric protein with one domain that binds to viral dsRNA and a second domain (e.g., a procaspase-binding domain or a procaspase) that induces apoptosis when two or more DRACOs crosslink on the same dsRNA. If viral dsRNA is present inside a cell, DRACOs will bind to the dsRNA and induce apoptosis of that cell. If viral dsRNA is not present inside the cell, DRACOs will not crosslink and apoptosis will not occur.

For delivery into cells *in vitro* or *in vivo*, DRACOs can be fused with proven protein transduction tags, including a sequence from the HIV TAT protein [28], the related protein transduction domain 4 (PTD) [29], and polyarginine (ARG) [30]. These tags have been shown to carry large cargo molecules into both the cytoplasm and the nucleus of all cell types *in vitro* and *in vivo*, even across the blood-brain barrier.

## Results and Discussion

**Figure 1:**
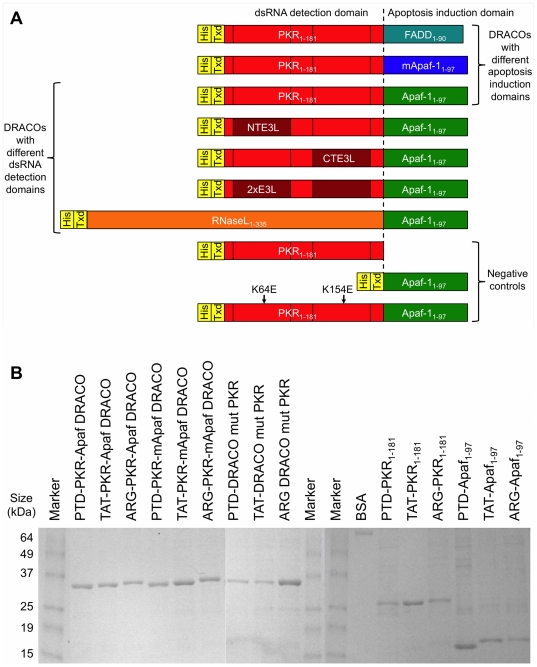
A variety of DRACOs and controls were produced. (**A**) DRACOs with different dsRNA detection and apoptosis induction domains were designed and produced. All domains were human except murine Apaf-1 (mApaf-1), and some dsRNA detection domains used PKR_1–181_ with vaccinia E3L dsRNA binding motif replacing PKR dsRBM 1 (NTE3L), dsRBM 2 (CTE3L), or both (2×E3L). His denotes His_6_ purification tag and Txd denotes PTD, TAT, or ARG transduction tag. DRACOs with transduction tags on the N-, C-, or both termini were produced. (**B**) This protein gel shows examples of DRACOs and negative controls that were produced. 1 µg was loaded per lane. Final yields were approximately 30 mg purified protein per liter of culture.

We produced DRACOs with different dsRNA detection domains, apoptosis induction domains, and transduction tags (Figure 1). The dsRNA detection domains included PKR_1–181_, PKR_1–181_ with dsRBM 1 (NTE3L), dsRBM 2 (CTE3L), or dsRBM 1 and 2 (2×E3L) replaced by the dsRNA binding motif from poxvirus E3L, and RNaseL_1–335_ (which binds to 2–5A produced by endogenous cellular 2–5A synthetases in response to viral dsRNA). The apoptosis induction domains included FADD_1–90_ Death Effector Domain (DED, which binds to procaspase 8), Apaf-1_1–97_ caspase recruitment domain (CARD, which binds to procaspase 9), and murine Apaf-1_1–97_ (mApaf) CARD. Except for mApaf, all domains refer to the human sequence. Isolated dsRNA detection domains and apoptosis induction domains were produced as negative controls. Mutant DRACOs with deleterious K64E [9] and homologous K154E mutations in the PKR domain were also produced as negative controls. Proteins were produced with TAT, PTD, or ARG tags on the N terminus, C terminus, or both termini. Proteins were expressed in BL21(DE3)pLysS Rosetta *E. coli*. An empty expression vector was transformed into the *E. coli* and the same purification protocol was followed, resulting in control extract without DRACOs.

**Figure 2:**
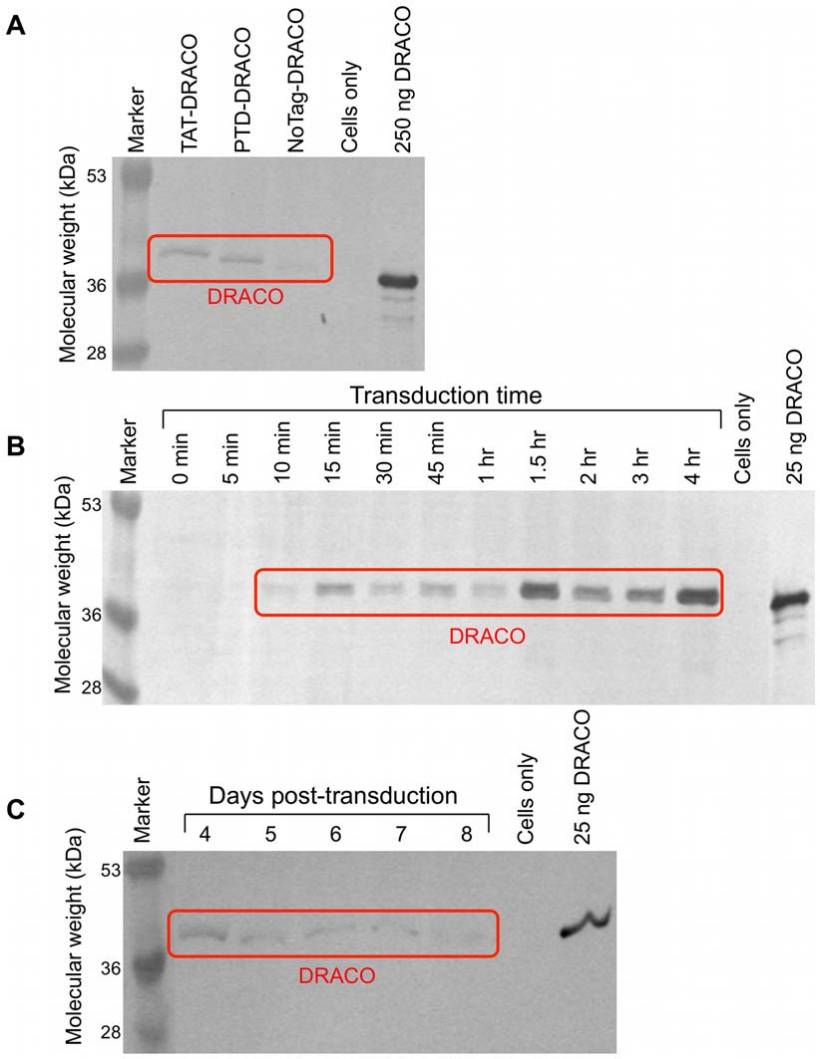
DRACOs penetrated cells and persisted for days. (**A**) DRACOs with PTD or TAT tags entered H1-HeLa cells more readily than DRACO without a transduction tag. 400 nM PKR-Apaf DRACO was added to medium for 1 hour, and then cells were trypsinized and washed to remove any DRACO on the cell surface. Cells were lysed and analyzed for DRACO by westerns using anti-His_6_ antibodies. Lysate from approximately 10^5^ cells was loaded in each lane. A known amount of purified PKR-Apaf DRACO was used as a standard as indicated. (**B**) DRACOs entered HeLa cells within 10 minutes and reached a maximum after 1.5 hours. 400 nM TAT-PKR-Apaf DRACO was added to medium for the specified time, and then cells were analyzed as in (A). (**C**) DRACOs persisted within HeLa cells for at least 8 days. 500 nM PTD-PKR-Apaf DRACO was added to cell medium for 1 hour, and then cells were put into DRACO-free medium. After the specified number of days, cells were analyzed as in (A).

**Figure 3:**
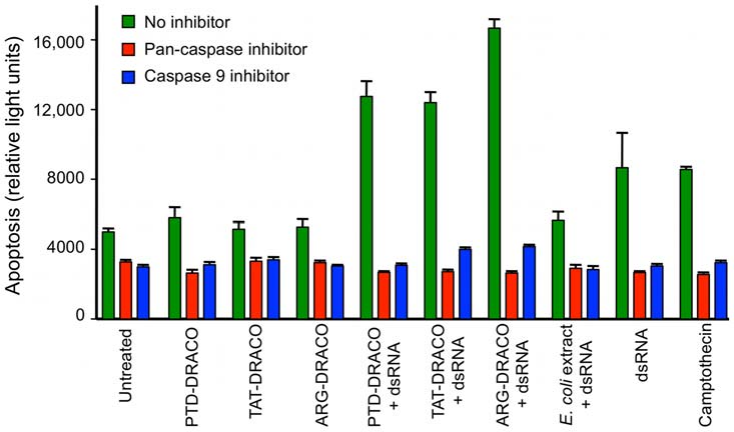
DRACOs mediated apoptosis in cells containing dsRNA. L929 cells transfected with both DRACO and poly(I)∶poly(C) dsRNA exhibited apoptosis within 24 hours, whereas cells that received only DRACO did not. Caspase inhibitors eliminated DRACO-mediated apoptosis in the presence of dsRNA.

DRACO rapidly entered cells, persisted within cells for days, and mediated apoptosis in cells transfected with dsRNA. PKR-Apaf DRACO with PTD or TAT tags entered cells efficiently, whereas DRACO without a transduction tag did not (Figure 2A). DRACO entered cells within 10 minutes, reached a maximum after approximately 1.5 hours (Figures 2B, S1), and persisted inside cells for at least 8 days (Figure 2C). L929 cells transfected with both DRACO and poly(I)∶poly(C) dsRNA exhibited greatly increased apoptosis within 24 hours, whereas cells that received only DRACO did not (Figure 3). Pan-caspase and caspase-9 inhibitors eliminated DRACO-mediated apoptosis in the presence of dsRNA.

**Figure 4:**
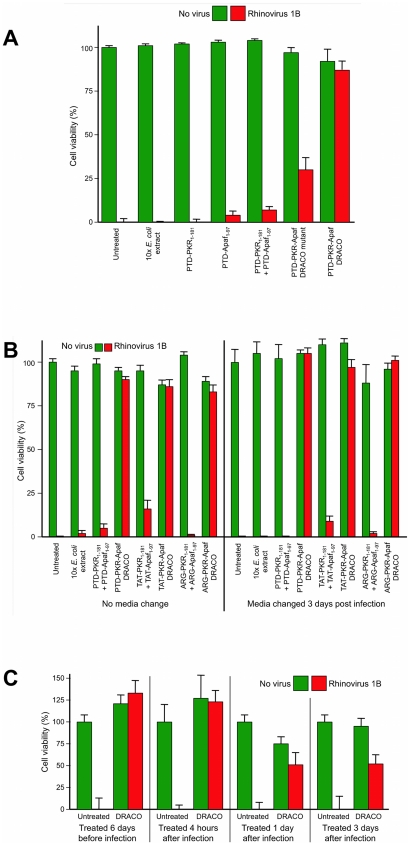
DRACOs were effective against rhinovirus 1B in NHLF cells. (**A**) 100 nM DRACO was effective against 130 pfu/well rhinovirus, whereas 100 nM negative controls were not (12 dpi). (**B**) Cell viability measured 7 dpi showed little difference if 100 nM DRACO-containing medium was removed 3 dpi when untreated cells had widespread CPE from 130 pfu/well rhinovirus 1B; there was no relapse of viral CPE in treated cells after DRACOs were withdrawn. (**C**) 1 dose of 25 nM PTD-PKR-Apaf DRACO was effective against rhinovirus 1B in NHLF cells when it was added from 6 days before infection to 3 days after infection. (Complete viral CPE in untreated cell populations required 3–4 days in our experiments, and for these experiments a significant fraction of cells were still uninfected 3 dpi.) Cell viability was measured 14 dpi.

We measured the viability of normal human lung fibroblast (NHLF) cells that had been treated with PKR-Apaf DRACOs or negative controls and then challenged with 130 plaque forming units (pfu) per well rhinovirus 1B (Figures 4, S2, S3). Untreated cell populations succumbed to virus within days, indicating that any innate cellular responses were ineffective against the virus or blocked by the virus. DRACOs with PTD, TAT, and ARG tags prevented significant cytopathic effects (CPE) in virus-challenged cell populations by rapidly killing any initially infected cells, thereby terminating the infection in its earliest stages. DRACOs had no apparent toxicity in unchallenged cells. Isolated PKR_1–181_ and Apaf-1_1–97_ domains were nontoxic but not antiviral, even when added simultaneously (but not covalently linked). DRACO with deleterious amino acid changes also had little efficacy. Likewise, an amount of purified bacterial extract (without DRACOs) approximately 10-fold greater than the average volume of DRACOs added to cells was nontoxic and not efficacious, demonstrating that any remaining bacterial contaminants such as lipopolysaccharide did not affect the cells or produce antiviral activity. Thus the antiviral efficacy appears to require intact functional DRACOs. Tests using DRACOs with protein transduction tags on the N terminus, C terminus, or both termini indicated that N-terminal tags generally worked the best (data not shown). DRACOs with transduction tags penetrated cells and were antiviral when administered by themselves (Figures 2, S2A), but efficacy was enhanced by co-administration with Roche FuGene 6 to maximize uptake (Figure S2B), so FuGene was used in experiments unless otherwise noted. Cell viability measured 7 days post infection (dpi) showed little difference if DRACO-containing medium was removed 3 dpi after untreated cells had widespread CPE; there was no relapse of viral CPE in treated cells after DRACOs were withdrawn (Figure 4B).

DRACOs were added approximately 24 hours before virus unless otherwise noted, but other dosing times were tested (Figure 4C). One dose of PTD-PKR-Apaf DRACO was efficacious against rhinovirus 1B in NHLF cells when added up to 6 days before infection, supporting the western data (Figure 2C) that DRACO persisted inside cells for at least 8 days. Up to 3 days after infection, one DRACO dose could still rescue a significant percentage of the cell population. After 3 days, virtually all of the cells had already been killed or at least infected by the virus.

**Figure 5:**
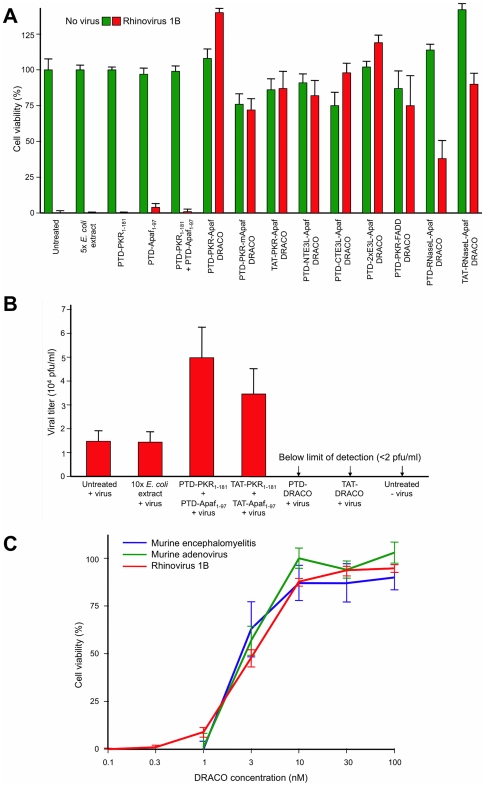
DRACOs were effective against rhinovirus 1B and other viruses. (**A**) Multiple 100 nM DRACOs were effective against 130 pfu/well rhinovirus (4 dpi). Even better performance of these alternate DRACOs might be achieved with further optimization. (**B**) PKR-Apaf DRACOs reduced the viral titer in supernatant from NHLF cells challenged with 300 pfu/well rhinovirus 1B to undetectable levels. PKR and Apaf-1 domains not covalently linked increased viral titers somewhat, possibly by interfering with the antiviral activity of endogenous wild-type PKR and Apaf-1. Cells were treated with 100 nM DRACO or controls. Supernatants were collected 4 dpi and their viral titers determined by serial dilution onto fresh 96-well NHLF plates. (**C**) The EC_50_ for PTD-PKR-Apaf DRACO was 2–3 nM against 130 pfu/well rhinovirus 1B in NHLF cells (measured 3 dpi), and 50 pfu/well murine encephalomyelitis (3 dpi) and 50 pfu/well murine adenovirus (11 dpi) in L929 cells.

Additional DRACO designs exhibited efficacy against rhinovirus (Figure 5A). Other effective dsRNA detection domains included NTE3L, CTE3L, 2×E3L, and RNaseL_1–335_. Other effective apoptotic domains included FADD_1–90_, mApaf1_1–97_, and procaspases [26]–[27]. Although the initial performance of these alternate DRACOs was generally inferior to that of PKR-Apaf human DRACO in these experiments, better performance might be achieved with further optimization. These results demonstrate that the alternate DRACO designs are nontoxic and efficacious against virus, and they support the DRACO mechanism of action.

In addition to improving survival of the cell population, DRACOs reduced viral titers from virus-challenged cells (Figures 5B, S4). One dose of PKR-Apaf DRACO administered to NHLF cells 24 hours before 300 pfu/well rhinovirus 1B eliminated any measurable viral titer in cell supernatant samples collected 4 dpi.

The median effective concentration for DRACOs with PTD, TAT, and ARG tags against a variety of viruses was 2–3 nM, as illustrated for PTD-PKR-Apaf DRACO against rhinovirus 1B, murine encephalomyelitis, and murine adenovirus (Figures 5C).

**Figure 6:**
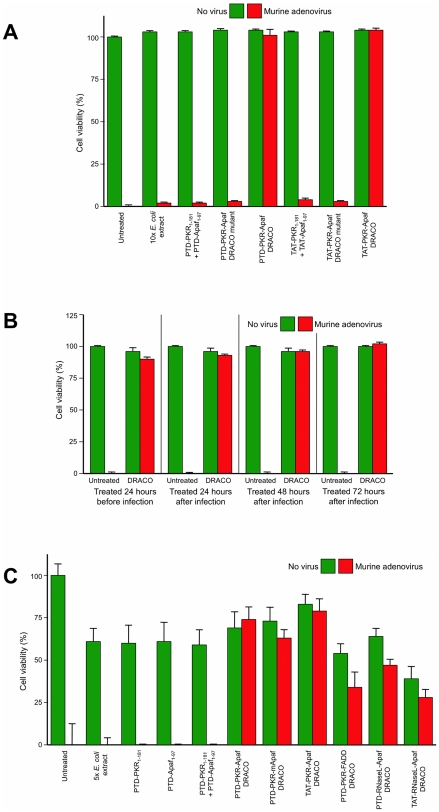
DRACOs were effective against murine adenovirus in L929 cells. (**A**) 100 nM DRACOs were effective against 50 pfu/well murine adenovirus, whereas all negative controls were not (16 dpi). (**B**) 100 nM PTD-PKR-Apaf DRACO was effective if added before or up to at least 72 hours after adenovirus (16 dpi). (**C**) Multiple 100 nM DRACOs were effective against 50 pfu/well murine adenovirus (11 dpi). Even better performance of these alternate DRACOs might be achieved with further optimization.

**Figure 7:**
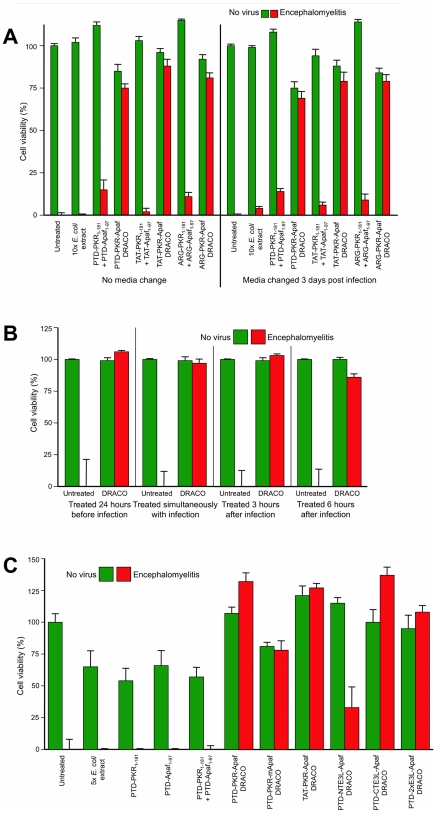
DRACOs were effective against murine encephalomyelitis in L929 cells. (**A**) 100 nM DRACOs were effective against 50 pfu/well encephalomyelitis. Cell viability measured 6 dpi showed little difference if DRACO-containing medium was removed 3 dpi when untreated cells had widespread CPE; there was no relapse of viral CPE in treated cells after DRACOs were withdrawn. (**B**) 100 nM PTD-PKR-Apaf DRACO was effective if added before, simultaneously with, or up to at least 6 hours after encephalomyelitis. (**C**) Multiple 100 nM DRACOs were effective against 50 pfu/well murine encephalomyelitis (4 dpi). Even better performance of these alternate DRACOs might be achieved with further optimization.

**Figure 8:**
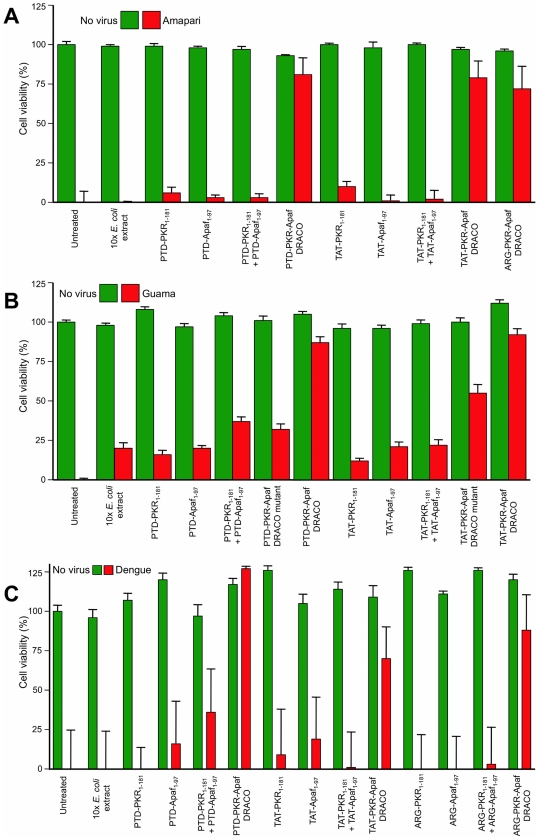
DRACOs were effective against arenaviruses, bunyaviruses, and flaviviruses. 200 nM DRACOs with PTD, TAT, and ARG protein transduction tags were effective in Vero E6 cells against (**A**) 30 pfu/well Amapari (assayed 15 dpi), (**B**) 30 pfu/well Guama strain Be An 277 (assayed 5 dpi), and (**C**) 10 pfu dengue type 2 (assayed 20 dpi).

DRACOs were effective against a broad spectrum of other viruses in a variety of cell types (Tables 1–2). DRACOs were effective against rhinoviruses 2 and 30 in NHLF cells (data not shown) and rhinovirus 14 in HeLa cells (Figure S4). DRACOs were effective against murine adenovirus in L929 cells if added before or up to at least 72 hours after virus (Figures 6, S5), demonstrating efficacy against a DNA virus (Figures 6A, S5), in murine cells (using human apoptotic DRACO domains to recruit endogenous murine procaspases), when treatment is delayed until significantly after infection (Figure 6B), and with a variety of DRACO designs (Figure 6C). DRACOs were effective against murine encephalomyelitis in L929 cells regardless of whether the DRACO-containing medium was removed 3 dpi (Figure 7A), whether DRACOs were added before or after infection (Figure 7B), and which DRACOs were used (Figures 7C, S6). DRACOs were effective in Vero E6 cells against Amapari and Tacaribe, arenaviruses that are closely related to lymphocytic choriomeningitis virus (LCMV), Lassa, and Junin viruses (Figures 8A, S7, S8). Likewise, DRACOs were effective against Guama strain Be An 277 (Figures 8B, S9); comparable results were obtained for Guama strain Be Ar 12590 (data not shown). Guama virus is a significant human pathogen and is closely related to other bunyaviruses such as Rift Valley fever, hantavirus, and Crimean-Congo virus. DRACOs were similarly effective against dengue type 2 (New Guinea C) hemorrhagic fever virus, a major human pathogen that is very closely related to other flaviviruses such as West Nile virus, Yellow fever virus, and Omsk virus (Figures 8C, S10, S11). DRACOs were also effective against H1N1 influenza A/PR/8/34 in normal human hepatocytes (Figure S12 left), reovirus 3 in BALB/3T3 murine cells (Figure S12 center), and adenovirus 5 in AD293 cells (Figure S12 right).

**Table 1:** We have demonstrated DRACO efficacy against a broad spectrum of viruses.

Virus	Family	Genome	Envelope	Replicates in	Species	Receptor
Rhinovirus 1B	Picornavirus	+ssRNA	No	Cytoplasm	Human	LDL receptor
Rhinovirus 2	Picornavirus	+ssRNA	No	Cytoplasm	Human	LDL receptor
Rhinovirus 14	Picornavirus	+ssRNA	No	Cytoplasm	Human	ICAM-1
Rhinovirus 30	Picornavirus	+ssRNA	No	Cytoplasm	Human	LDL receptor
Theiler's encephalomyelitis	Picornavirus	+ssRNA	No	Cytoplasm	Mouse	Sialic acid
Dengue type 2	Flavivirus	+ssRNA	Yes	Cytoplasm	Human	DC-SIGN, etc.
Influenza H1N1 A/PR/8/34	Orthomyxovirus	−ssRNA	Yes	Nucleus	Human	Sialic acid
Influenza H1N1 A/WS/33	Orthomyxovirus	−ssRNA	Yes	Nucleus	Human	Sialic acid
Tacaribe	Arenavirus	−ssRNA	Yes	Cytoplasm	Bat	Transferrin receptor 1
Amapari	Arenavirus	−ssRNA	Yes	Cytoplasm	Rodent	Transferrin receptor 1
Guama Be An 277	Bunyavirus	−ssRNA	Yes	Cytoplasm	Rodent	Unidentified
Guama Be Ar 12590	Bunyavirus	−ssRNA	Yes	Cytoplasm	Rodent	Unidentified
Reovirus 3	Reovirus	dsRNA	No	Cytoplasm	Human	Sialic acid
Adenovirus 5	Adenovirus	dsDNA	No	Nucleus	Human	CAR
Murine adenovirus	Adenovirus	dsDNA	No	Nucleus	Mouse	CAR

These include viruses with DNA, dsRNA, positive-sense ssRNA, and negative-sense ssRNA genomes; enveloped and non-enveloped viruses; viruses that replicate in the cytoplasm and viruses that replicate in the nucleus; human, bat, and rodent viruses; and viruses that use a variety of cellular receptors.

**Table 2:** We have demonstrated that DRACO is effective and nontoxic in a wide variety of cell types.

Cells	Species	Tissue	Immortalized	Viruses
Lung fibroblasts	Human	Lung	No	Rhino 1B, 2, 30; Flu 33, 34
Hepatocytes	Human	Liver	No	Rhino 1B, 2, 30; Flu 33, 34
Airway epithelial	Human	Trachea	No	Flu A/PR/8/34
Osteoblasts	Human	Bone	No	Rhino 1B, 2, 30; Flu 33, 34
Aortic muscle	Human	Heart	No	Rhino 1B, 2, 14, 30; Flu 33, 34
AD293	Human	Kidney	Yes	Adeno 5
H1-HeLa	Human	Cervix	Yes	Rhino 14
Vero E6	Monkey	Kidney	Yes	Amapari, Tacaribe, Guama, Dengue
L929	Mouse	Fibroblast	Yes	Enceph, MAdeno, Reo 3
BALB/3T3	Mouse	Fibroblast	Yes	Reo 3
NIH/3T3	Mouse	Fibroblast	Yes	Encephalomyelitis

These include cells representing a variety of tissues; human, mouse, and monkey cells, and both primary and immortalized cells.

**Figure 9:**
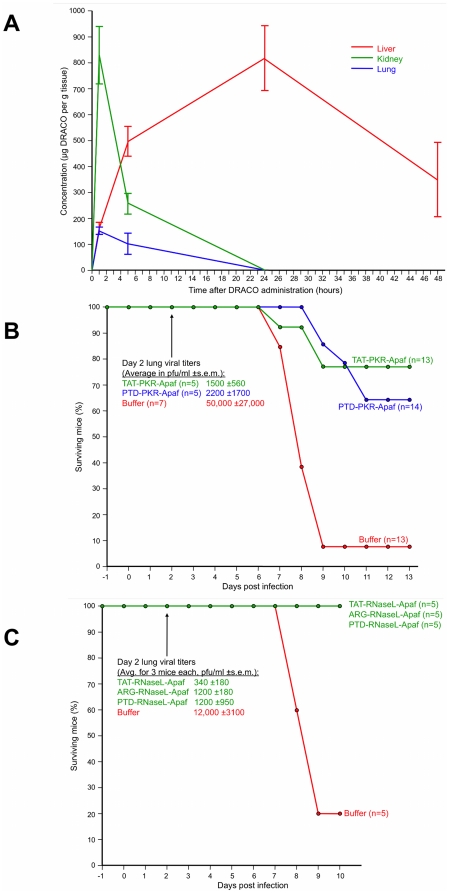
DRACOs appeared promising when administered via intraperitoneal (i.p.) injection in proof-of-concept trials with adult BALB/c mice. (**A**) 2.5 mg PTD-PKR-Apaf DRACO administered i.p. penetrated the liver, kidney, and lungs and persisted for at least 48 hours. Averages of 3 mice per data point are plotted, and error bars show s.e.m. (**B**) PTD-PKR-Apaf and TAT-PKR-Apaf DRACOs administered i.p. from day -1 through day 3 greatly reduced the morbidity and day-2 lung viral titers in mice challenged intranasally (i.n.) with 1.3 LD_50_ influenza H1N1 A/PR/8/34. (**C**) PTD-RNaseL-Apaf, TAT-RNaseL-Apaf, and ARG-RNaseL-Apaf DRACOs administered i.p. from day -1 through day 3 greatly reduced the morbidity and day-2 lung viral titers in mice challenged i.n. with 0.3 LD_50_ influenza H1N1 A/PR/8/34.

**Figure 10:**
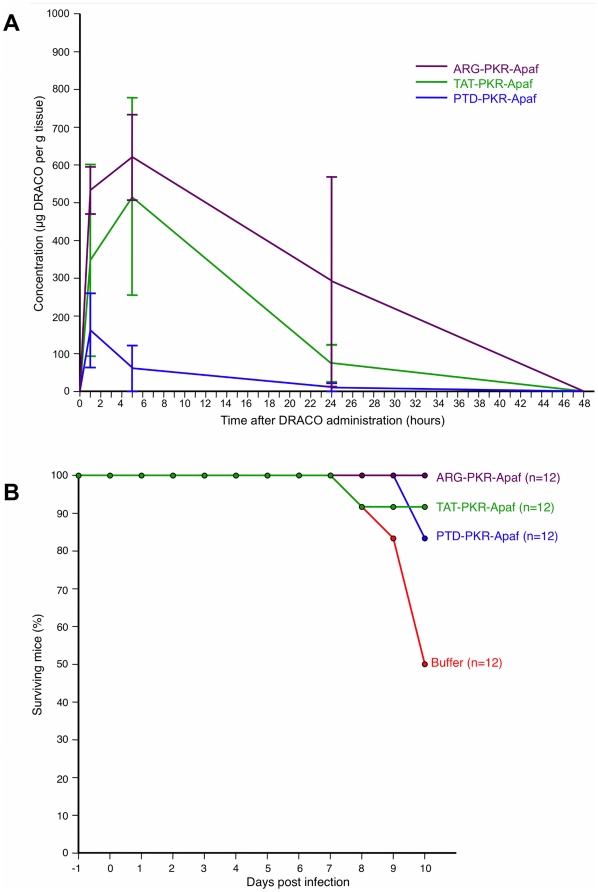
DRACOs appeared promising when administered via intranasal (i.n.) injection in proof-of-concept trials with adult BALB/c mice. (**A**) 0.5 mg PKR-Apaf DRACO administered i.n. to adult BALB/c mice penetrated the lungs and persisted over 24 hours. Averages of 3 mice per data point are plotted, and error bars show s.e.m. (**B**) PTD-PKR-Apaf, TAT-PKR-Apaf, and ARG-PKR-Apaf DRACOs administered i.n. on day 0 reduced the morbidity in mice challenged i.n. with 1 LD_50_ influenza H1N1 A/PR/8/34.

DRACOs appeared promising in proof-of-concept trials with adult BALB/c mice. Intraperitoneal (i.p.) PKR-Apaf DRACO penetrated the liver, kidney, and lungs and persisted at least 24–48 hours (Figure 9A). Live mice and harvested mouse organs showed no apparent toxicity. PTD-PKR-Apaf and TAT-PKR-Apaf DRACOs administered i.p. from day -1 through day 3 greatly reduced the morbidity in mice challenged intranasally (i.n.) with 1.3 LD_50_ influenza H1N1 A/PR/8/34 and reduced the day-2 lung viral titers by over an order of magnitude (Figure 9B). Similarly, PTD-RNaseL-Apaf, TAT-RNaseL-Apaf, and ARG-RNaseL-Apaf DRACOs administered i.p. from day -1 through day 3 prevented morbidity in mice challenged i.n. with 0.3 LD_50_ influenza and reduced the day-2 viral titers by an order of magnitude or more (Figure 9C). PKR-Apaf DRACO administered i.n. to mice penetrated the lungs and persisted over 24 hours (Figure 10A). PTD-PKR-Apaf, TAT-PKR-Apaf, and ARG-PKR-Apaf DRACOs administered i.n. on day 0 reduced the morbidity in mice challenged i.n. with 1 LD_50_ influenza (Figure 10B).

Based on these encouraging initial animal trials, future work should be done to test and optimize antiviral efficacy, pharmacokinetics, and absence of toxicity *in vitro* and *in vivo*. Future experiments can further characterize and optimize dsRNA binding, apoptosis induction, cellular transduction, and other DRACO properties. More extensive trials are also needed to determine how long after infection DRACOs can be used successfully, or if DRACOs are useful against chronic viral infections without producing unacceptable levels of cell death *in vivo*.

DRACOs should be effective against numerous clinical and NIAID priority viruses, due to the broad-spectrum sensitivity of the dsRNA detection domain, the potent activity of the apoptosis induction domain, and the novel direct linkage between the two which viruses have never encountered. We have demonstrated that DRACOs are effective against viruses with DNA, dsRNA, positive-sense ssRNA, and negative-sense ssRNA genomes; enveloped and non-enveloped viruses; viruses that replicate in the cytoplasm and viruses that replicate in the nucleus; human, bat, and rodent viruses; and viruses that use a variety of cellular receptors (Table 1).

## Materials and Methods

### Ethics statement for mouse trials

This study was carried out in strict accordance with the recommendations in the Guide for the Care and Use of Laboratory Animals of the National Institutes of Health. The protocol was approved by the Committee on Animal Care of MIT (Assurance Number: A-3125-01). Guidelines to minimize suffering were followed, and avertin anesthetic was used for intranasal procedures.

### Cloning

E.F. Meurs provided PKR cDNA and Y. Shi donated human Apaf-1 cDNA. RNaseL_1–335_ sequence was cloned from HeLa cells. PKR-E3L, FADD_1–90_ (with L75A and L76A to prevent spontaneous self-association [18]) and murine Apaf-1_1–97_ sequences came from BioBasic. Genes for DRACOs and controls were constructed using PCR and restriction cloning. TAT (YGRKKRRQRRR), PTD-4 (YARAAARQARA), and ARG (R_9_) tags were incorporated at N- and/or C-termini. Genes were inserted into pET100/D-TOPO (Invitrogen).

### Protein production

Each vector was transformed into Rosetta BL21(DE3)pLysS *E. coli* (EMD Biosciences), bacteria were plated on Luria broth (LB) agar with 100 µg ml^−1^ ampicillin and 34 µg ml^−1^ chloramphenicol, and plates were incubated overnight at 37°C. One colony was inoculated into ampicillin-chloramphenicol LB and grown overnight (37°C, 225 r.p.m.), then diluted 1∶30 into ampicillin-chloramphenicol LB and incubated (30°C, 225 r.p.m.) until OD600 reached 1.0. 0.5 mM isopropyl β-D-1-thiogalactopyranoside was added and flasks were incubated overnight. *E. coli* were recovered by centrifugation (5,000 r.p.m., 30 min., 4°C) and lysed by sonication, and His_6_-tagged proteins were purified using Ni-NTA agarose (Invitrogen) following the manufacturer's protocols. Proteins were eluted into 1.5× PBS with 300 mM imidazole and 10% (vol/vol) glycerol, concentrated with Amicon-15 (10 kDa cutoff, 3,000 *g*) to >5 mg/ml, and filter-sterilized. Protein concentrations were measured relative to BSA standards by Bradford assay (BioRad) and Gel Doc densitometry.

### Cells

L929 (CCL-1), NIH/3T3 (CRL-1658), BALB/3T3 (CCL-163), H1-HeLa (CRL-1958), MDCK (CCL-34), and Vero E6 (CRL-1586) (ATCC) and AD293 (Stratagene) were cultured in complete DMEM (Gibco). Normal human lung fibroblasts, small airway epithelial cells, osteoblasts, hepatocytes, and aortic smooth muscle cells (Lonza) were cultured in cell-specific media (Lonza).

### Viruses

Dengue type 2 (New Guinea C, VR-1584), Amapari (VR-477), Tacaribe (VR-1272), Guama (Be An 277, VR-407; Be Ar 12590, VR-420), murine adenovirus (VR-550), Theiler's murine encephalomyelitis (VR-57), reovirus 3 (VR-824), influenza H1N1 A/PR/8/34 (ATCC VR-1469), influenza H1N1 A/WS/33 (ATCC VR-1520), rhinovirus 1B (VR-481), rhinovirus 2 (VR-482), rhinovirus 14 (VR-284), and rhinovirus 30 (VR-505) were obtained from ATCC. Adenovirus 5 was obtained from Stratagene. Influenza A/PR/8/34 for animal trials was donated by P. Palese.

### Cell assays

Contact-inhibited cells were grown to 50–80% confluence and non-contact inhibited cells to 20–50% confluence in 96-well plates with 100 µl/well medium. DRACOs or controls were added to columns of wells, 8 wells/column. Except in Figs. 2 and S2, 0.4–1% (vol/vol) Roche FuGene 6 was co-administered with DRACOs and controls to optimize cellular uptake. Wells received virus approximately 24 hours after DRACO unless otherwise noted. On selected days, cell viability in each plate was measured using CellTiter 96 (Promega). Assay schedules, viral doses, and other parameters were optimized for different cell/virus systems. Micrographs were taken in 24-well plates under similar conditions.

### DRACO cell penetration assays

Cells in 24-well plates were incubated with DRACOs for varying lengths of time, then trypsinized, washed thoroughly in PBS, and lysed. Lysate from approximately 10^5^ cells was loaded in each lane. DRACOs were detected via westerns using mouse anti-His_6_ (Invitrogen) and goat anti-mouse IgG HRP (Jackson).

### Apoptosis assays

70% confluent 96-well L929 plates were treated with 10 µM Z-VAD-FMK pan-caspase inhibitor or 20 µM Z-LEHD-FMK caspase-9 inhibitor (R&D Systems), then 75 µM camptothecin (Calbiochem) or 100 nM DRACOs with or without 25 ng/well poly(I)∶poly(C) dsRNA (Sigma) transfected using FuGene (Roche) following manufacturers' protocols. After 24 hours, apoptosis was determined using Caspase-Glo 3/7 (Promega).

### Viral titers

Titers were determined by serial dilutions onto 96-well NHLF (for rhinovirus 1B) or H1-HeLa (for rhinovirus 14) plates, with 8 wells per 10-fold dilution and with the number of wells exhibiting CPE measured 5 dpi. Reed-Muench titers were calculated from the results (1 TCID_50_≈0.7 pfu). Error bars indicate s.e.m. from 3 trials.

### Statistical analysis

CellTiter 96 cell viabilities were normalized to 100% for untreated uninfected and 0% for untreated virus-killed cells. Graphs indicate averages (n = 8) with s.e.m. Experiments were repeated at least 3 times with similar results.

### Mouse trials

7-week-old female BALB/c mice (Charles River) received DRACO i.n. (∼0.5 mg in 50 µl) or i.p. (0.8–2.5 mg in 200 µl). Mice were challenged i.n. with 0.3–1.3 LD_50_ influenza H1N1 A/PR/8/34. Mice received DRACO i.p. once daily on days -1 and 1–3 and twice on day 0, or just one i.n. DRACO dose simultaneously with virus. Lungs were harvested on day 2 and viral titers determined by serial dilutions onto 96-well MDCK plates. For pharmacokinetics, organs were harvested at designated times, then sonicated into 1 ml PBS with 1% Triton X-100. 1 mg organ solution was mixed with 2× Laemmeli buffer, boiled 5 min., and run on a 10–20% SDS PAGE gel with a standard curve of purified DRACO, followed by western blots with anti-Apaf (Millipore) and HEP-labeled anti-rabbit IgG (Jackson Immunoresearch). Blots were developed with Pierce luminescent reagent and exposed to film. DRACO bands were quantitated by Gel Doc densitometry vs. the standards.

## Supporting Information

Figure S1 **DRACOs entered normal human lung fibroblasts.** NHLF cells were incubated overnight with 500 nM PTD-PKR-Apaf DRACO labeled with Lumio (Invitrogen), washed with Hank's balanced salt solution, and photographed with a fluorescent microscope to compare (**A**) untreated and (**B**) DRACO-treated cells. DRACOs appeared to be distributed throughout each cell in both the cytoplasm and the nucleus.
(TIF)

Figure S2 **FuGene co-administration with DRACOs improved cellular uptake and antiviral efficacy.** (**A**) 100 nM DRACOs with PTD, TAT, and ARG protein transduction tags were effective against rhinovirus 1B in NHLF cells without FuGene co-administration. Cell viability was measured 3 days after infection with 130 pfu/well. (**B**) Co-administration of FuGene with DRACOs lowered the EC_50_ of DRACOs, as shown here for PTD-PKR Apaf DRACO against 130 pfu/well rhinovirus 1B in NHLFs.
(TIF)

Figure S3 **200 nM PTD-PKR-Apaf DRACO was effective against rhinovirus 1B in NHLF cells.** Representative photographs were taken 20 days after challenge with 300 pfu/well. Scale bar = 50 µm.
(TIF)

Figure S4 **DRACOs decreased the viral titer of rhinovirus 14 in H1-HeLa cells.** One 120 nM dose of PTD-PKR-Apaf DRACO administered to cells 24 hours before or simultaneously with 10 pfu/well rhinovirus 14 eliminated any measurable titer 3 dpi. One DRACO dose administered 24 or 30 hours after infection halved the 3-dpi viral titer.
(TIF)

Figure S5 **200 nM PTD-PKR-Apaf DRACO was effective against murine adenovirus in L929 cells.** Representative photographs were taken 15 days after challenge with 30 pfu/well. Scale bar = 25 µm.
(TIF)

Figure S6 **200 nM PTD-PKR-Apaf DRACO was effective against murine encephalomyelitis in L929 cells.** Representative photographs were taken 21 days after challenge with 50 pfu/well. Scale bar = 25 µm.
(TIF)

Figure S7 **100 nM PTD-PKR-Apaf DRACO was effective against Amapari arenavirus in Vero E6 cells.** Representative photographs were taken 11 days after challenge with 300 pfu/well. Scale bar = 100 µm.
(TIF)

Figure S8 **100 nM PTD-PKR-Apaf DRACO was effective against Tacaribe arenavirus in Vero E6 cells.** Photographs were taken 8 days after challenge with 140 pfu/well. Scale bar = 100 µm.
(TIF)

Figure S9 **200 nM PTD-PKR-Apaf DRACO was effective against Guama Be An 277 bunyavirus in Vero E6 cells.** Photographs were taken 4 days after challenge with 30 pfu/well. Scale bar = 100 µm.
(TIF)

Figure S10 **200 nM PKR-Apaf DRACO was effective against dengue flavivirus in Vero E6 cells.** Cell viability was measured 18 days after challenge with 16 pfu/well.
(TIF)

Figure S11 **100 nM PTD-PKR-Apaf DRACO was effective against dengue flavivirus in Vero E6 cells.** Photographs were taken 7 days after challenge with 160 pfu/well. Scale bar = 100 µm.
(TIF)

Figure S12 **DRACOs were effective against a broad spectrum of other viruses in a variety of cell types.** Left four photos: 100 nM PKR-Apaf DRACO was effective against H1N1 influenza A/PR/8/34 in normal human hepatocytes. Untreated cells challenged with 10^5^ pfu/well died within 3 days, whereas treated challenged cells were cultured for 72 days with no sign of viral CPE. Center four photos: 100 nM PTD-PKR-Apaf DRACO was effective against reovirus 3 in BALB/3T3 murine cells. Photographs were taken 11 days after challenge with 30 pfu/well reovirus 3. Right four photos: 200 nM PTD-2×E3L-Apaf DRACO was effective against adenovirus 5 in human embryonic kidney AD293 cells. Fluorescent microscope photographs were taken 4 days after challenge with 25 pfu/well adenovirus 5 expressing enhanced green fluorescent protein (EGFP). Scale bars = 50 µm.
(TIF)

## References

[1] Kräusslich HG, Bartenschlager R (2009). Handbook of Experimental Pharmacology 189: Antiviral Strategies.

[2] Demberg T, Robert-Guroff M (2009). Mucosal immunity and protection against HIV/SIV infection: strategies and challenges for vaccine design.. Int Rev Immunol.

[3] Boomker JM, de Leij LF, The TH, Harmsen MC (2005). Viral chemokine-modulatory proteins: tools and targets.. Cytokine Growth Factor Rev.

[4] Knipe DM, Howley PM (2006). Fields Virology, 5th ed.

[5] Samuel CE (2004). Knockdown by RNAi—proceed with caution.. Nat Biotechnol.

[6] Sadler AJ, Williams BRG (2008). Interferon-inducible antiviral effectors.. Nat Rev Immunol.

[7] Yoneyama M, Fujita T (2010). Recognition of viral nucleic acids in innate immunity.. Rev Med Virol.

[8] Sadler AJ, Williams BR (2007). Structure and function of the protein kinase R.. Curr Top Microbiol Immunol.

[9] Wu S, Kaufman RJ (1996). Double-stranded (ds) RNA binding and not dimerization correlates with the activation of the dsRNA-dependent protein kinase (PKR).. J Biol Chem.

[10] Hovanessian AG, Justesen J (2007). The human 2′-5′oligoadenylate synthetase family: unique interferon-inducible enzymes catalyzing 2′-5′ instead of 3′-5′ phosphodiester bond formation.. Biochimie.

[11] Bisbal C, Silverman RH (2007). Diverse functions of RNase L and implications in pathology.. Biochimie.

[12] Kawai T, Akira S (2010). The role of pattern-recognition receptors in innate immunity: update on Toll-like receptors.. Nat Immunol.

[13] Placido D, Brown BA, Lowenhaupt K, Rich A, Athanasiadis A (2007). A left-handed RNA double helix bound by the Zα domain of the RNA-editing enzyme ADAR1.. Structure.

[14] Pop C, Salvesen GS (2009). Human caspases: activation, specificity, and regulation.. J Biol Chem.

[15] Qin H, Srinivasula SM, Wu G, Fernandes-Alnemri T, Alnemri ES (1999). Structural basis of procaspase-9 recruitment by the apoptotic protease-activating factor 1.. Nature.

[16] Bao Q, Shi Y (2007). Apoptosome: a platform for the activation of initiator caspases.. Cell Death Differ.

[17] Eberstadt M, Huang B, Chen Z, Meadows RP, Ng SC (1998). NMR structure and mutagenesis of the FADD (Mort1) death-effector domain.. Nature.

[18] Muppidi JR, Lobito AA, Ramaswamy M, Yang JK, Wang L (2006). Homotypic FADD interactions through a conserved RXDLL motif are required for death receptor-induced apoptosis.. Cell Death Differ.

[19] Randall RE, Goodbourn S (2008). Interferons and viruses: an interplay between induction, signalling, antiviral responses and virus countermeasures.. J Gen Virol.

[20] Weber F, Haller O (2007). Viral suppression of the interferon system.. Biochimie.

[21] Langland JO, Kash JC, Carter V, Thomas MJ, Katze MG (2006). Suppression of proinflammatory signal transduction and gene expression by the dual nucleic acid binding domains of the vaccinia virus E3L proteins.. J Virol.

[22] Galluzzi L, Brenner C, Morselli E, Touat Z, Kroemer G (2008). Viral control of mitochondrial apoptosis.. PLoS Pathogens.

[23] Postigo A, Ferrer PE (2009). Viral inhibitors reveal overlapping themes in regulation of cell death and innate immunity.. Microbes Infect.

[24] Nogal ML, González de Buitrago G, Rodríguez C, Cubelos B, Carrascosa AL (2001). African swine fever virus IAP homologue inhibits caspase activation and promotes cell survival in mammalian cells.. J Virol.

[25] Callus BA, Vaux DL (2007). Caspase inhibitors: viral, cellular and chemical.. Cell Death Differ.

[26] Rider TH (2006). Anti-pathogen treatments..

[27] Rider TH (2009). Anti-pathogen treatments..

[28] Gump JM, Dowdy SF (2007). TAT transduction: the molecular mechanism and therapeutic prospects.. Trends Mol Med.

[29] Ho A, Schwarze SR, Mermelstein SJ, Waksman G, Dowdy SF (2001). Synthetic protein transduction domains: enhanced transduction potential in vitro and in vivo.. Cancer Res.

[30] Goun EA, Pillow TH, Jones LR, Rothbard JB, Wender PA (2006). Molecular transporters: synthesis of oligoguanidinium transporters and their application to drug delivery and real-time imaging.. ChemBioChem.

