# Supplementary figures and images for: Broad-Spectrum Antiviral Therapeutics
Source: PLoS One. 2011 Jul 27;6(7):e22572. doi: 10.1371/journal.pone.0022572 (PMC3144912; doi:10.1371/journal.pone.0022572)

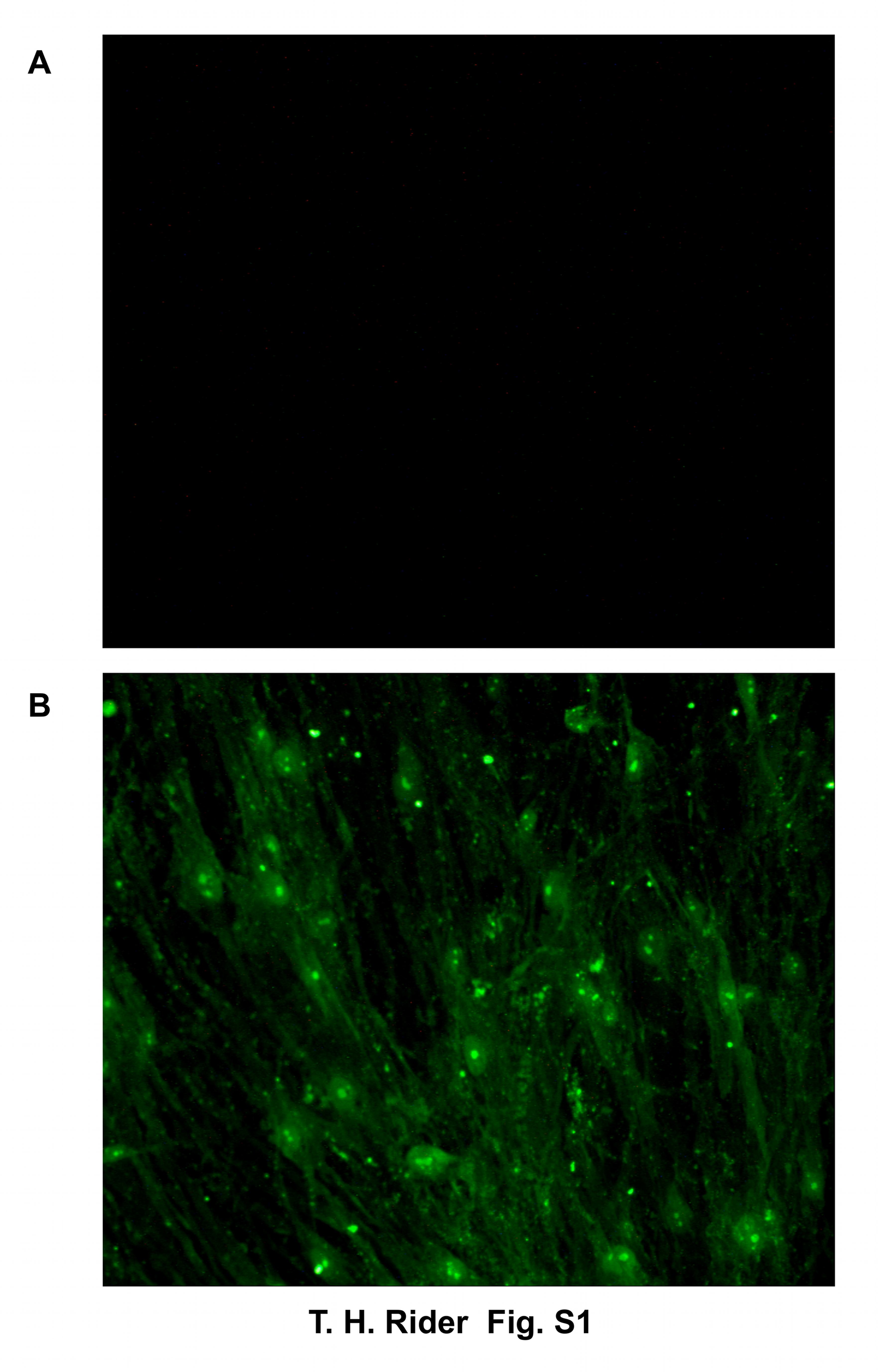

Supplement: Figure S1 — DRACOs entered normal human lung fibroblasts. NHLF cells were incubated overnight with 500 nM PTD-PKR-Apaf DRACO labeled with Lumio (Invitrogen), washed with Hank's balanced salt solution, and photographed with a fluorescent microscope to compare (A) untreated and (B) DRACO-treated cells. DRACOs appeared to be distributed throughout each cell in both the cytoplasm and the nucleus. (TIF) [file pone.0022572.s001.tif]

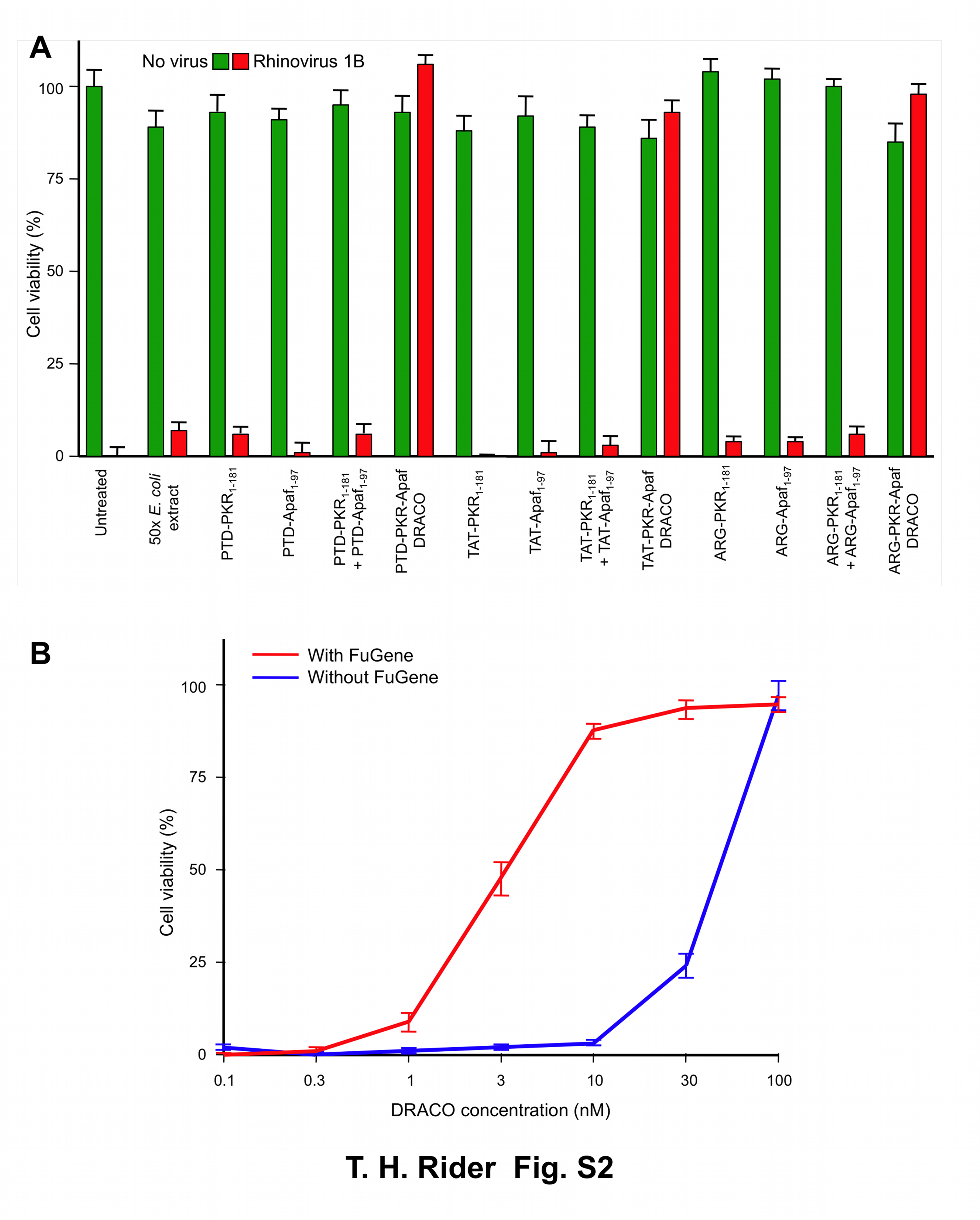

Supplement: Figure S2 — FuGene co-administration with DRACOs improved cellular uptake and antiviral efficacy. (A) 100 nM DRACOs with PTD, TAT, and ARG protein transduction tags were effective against rhinovirus 1B in NHLF cells without FuGene co-administration. Cell viability was measured 3 days after infection with 130 pfu/well. (B) Co-administration of FuGene with DRACOs lowered the EC50 of DRACOs, as shown here for PTD-PKR Apaf DRACO against 130 pfu/well rhinovirus 1B in NHLFs. (TIF) [file pone.0022572.s002.tif]

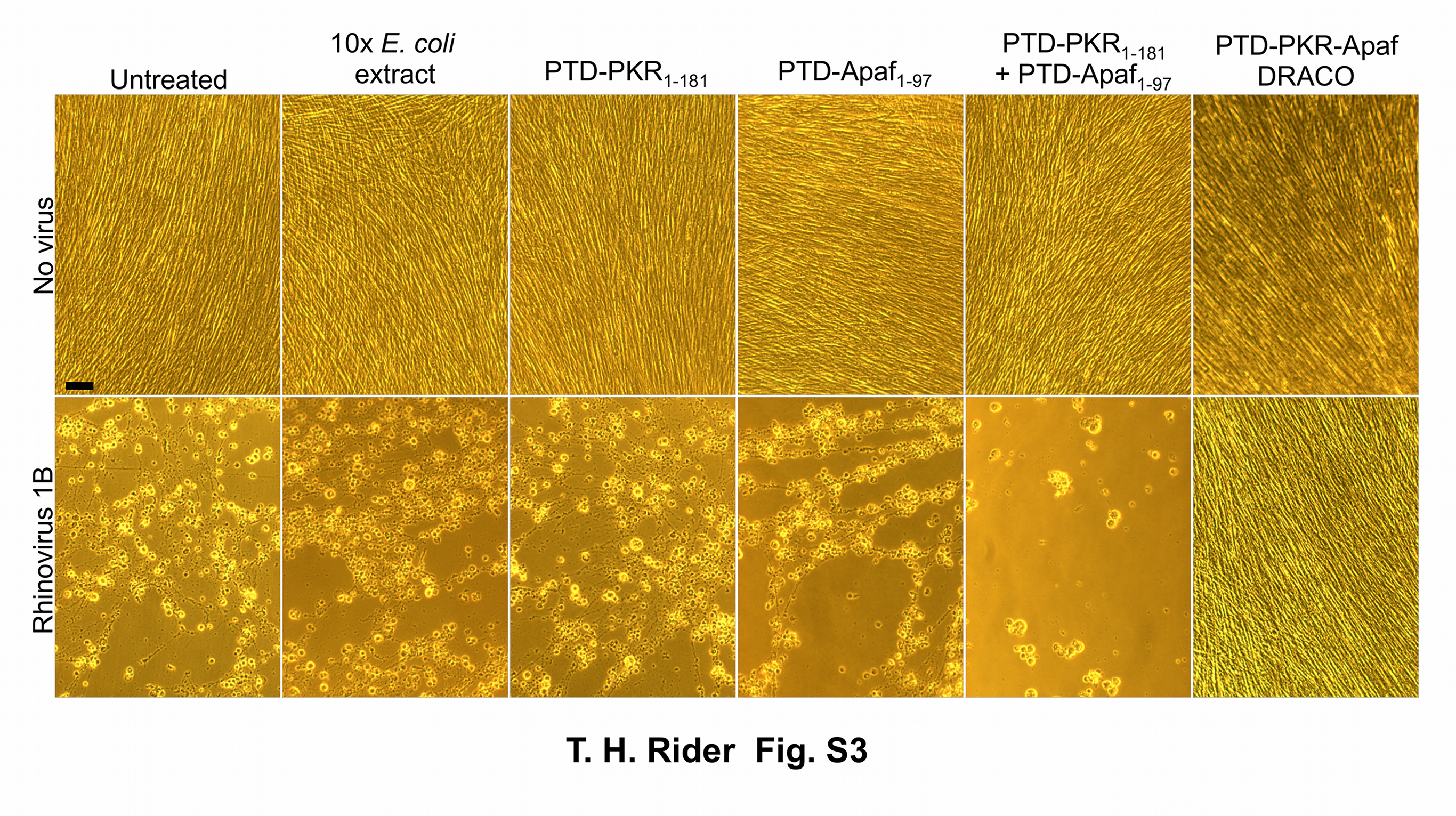

Supplement: Figure S3 — 200 nM PTD-PKR-Apaf DRACO was effective against rhinovirus 1B in NHLF cells. Representative photographs were taken 20 days after challenge with 300 pfu/well. Scale bar = 50 µm. (TIF) [file pone.0022572.s003.tif]

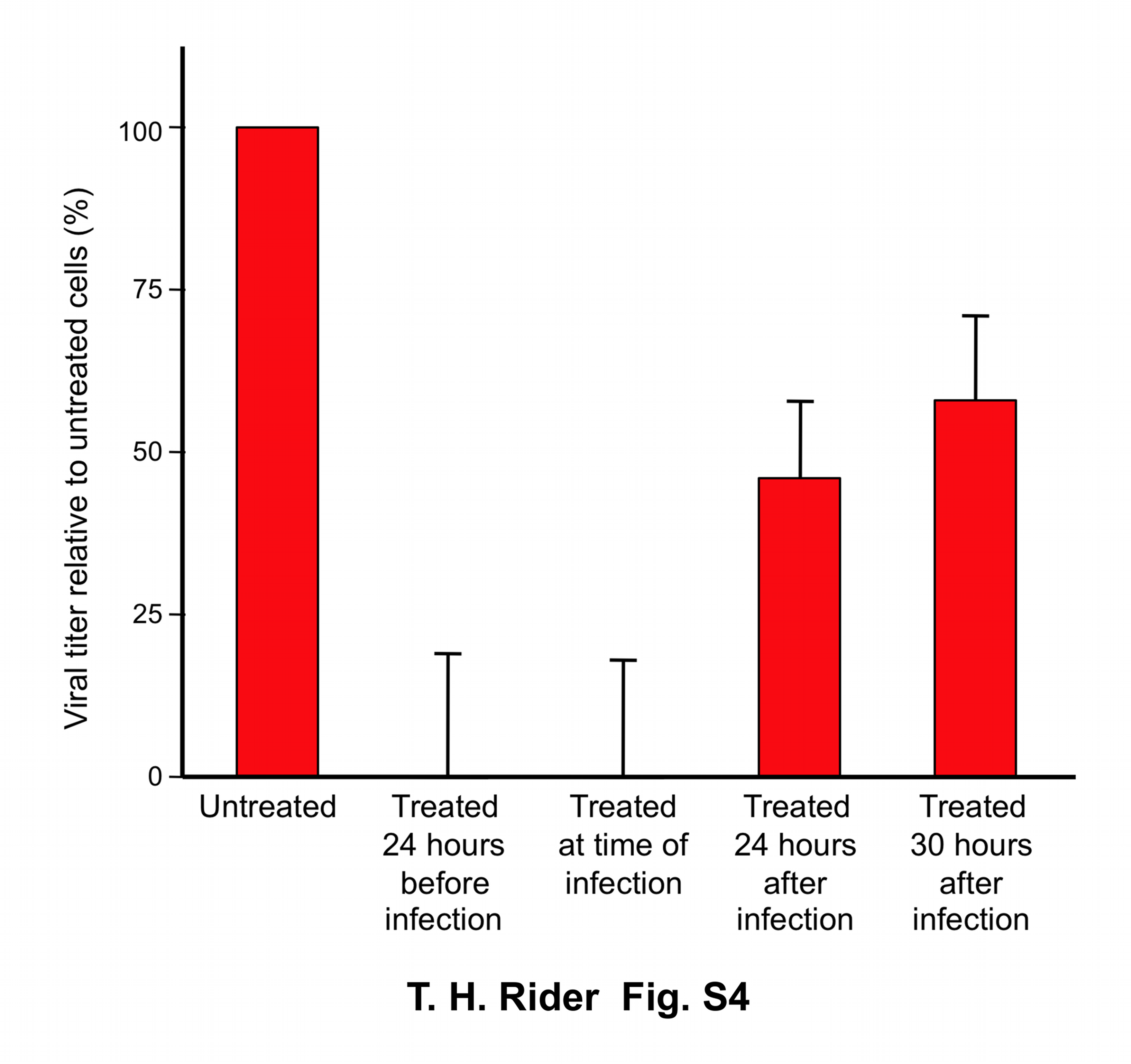

Supplement: Figure S4 — DRACOs decreased the viral titer of rhinovirus 14 in H1-HeLa cells. One 120 nM dose of PTD-PKR-Apaf DRACO administered to cells 24 hours before or simultaneously with 10 pfu/well rhinovirus 14 eliminated any measurable titer 3 dpi. One DRACO dose administered 24 or 30 hours after infection halved the 3-dpi viral titer. (TIF) [file pone.0022572.s004.tif]

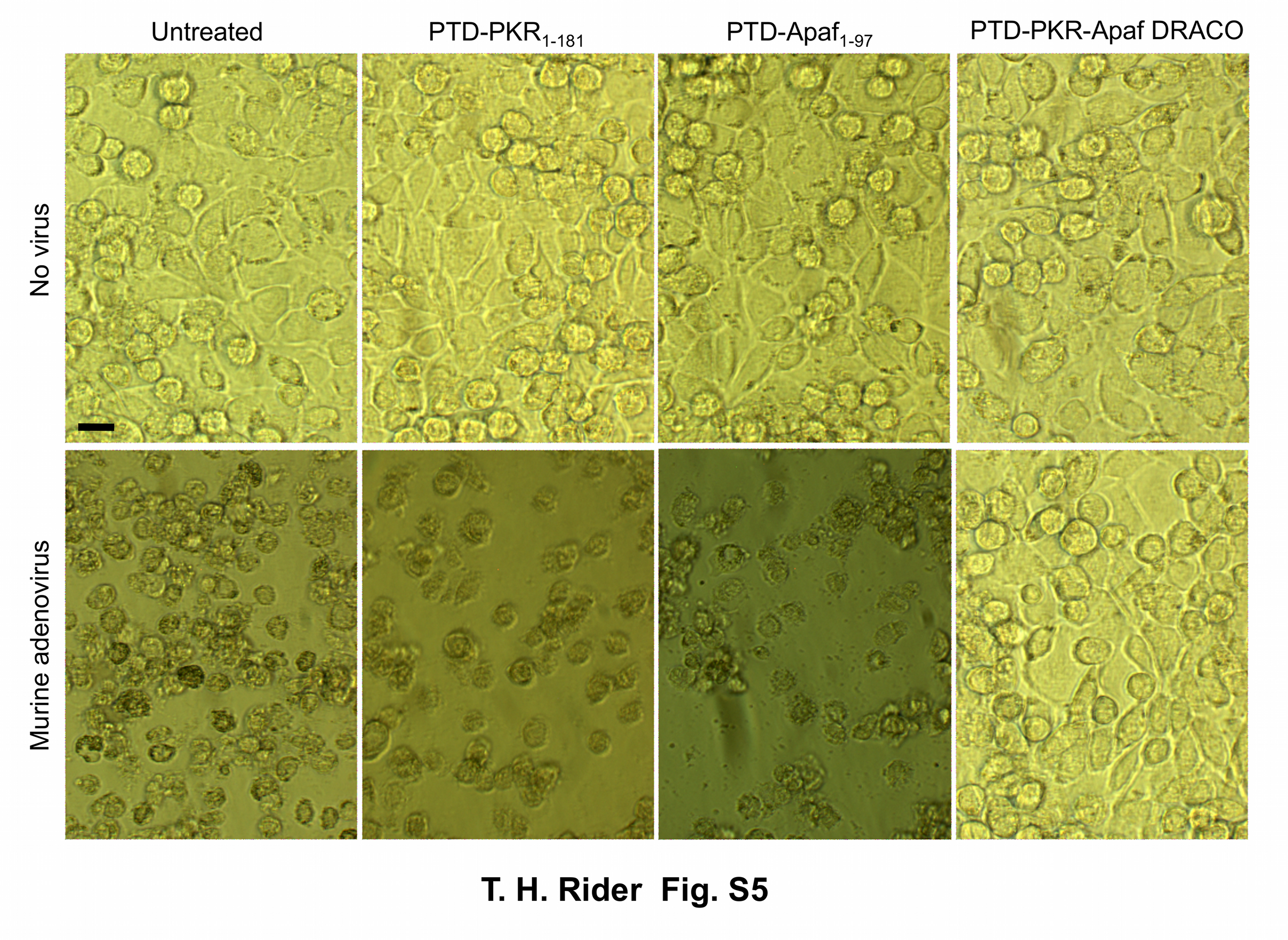

Supplement: Figure S5 — 200 nM PTD-PKR-Apaf DRACO was effective against murine adenovirus in L929 cells. Representative photographs were taken 15 days after challenge with 30 pfu/well. Scale bar = 25 µm. (TIF) [file pone.0022572.s005.tif]

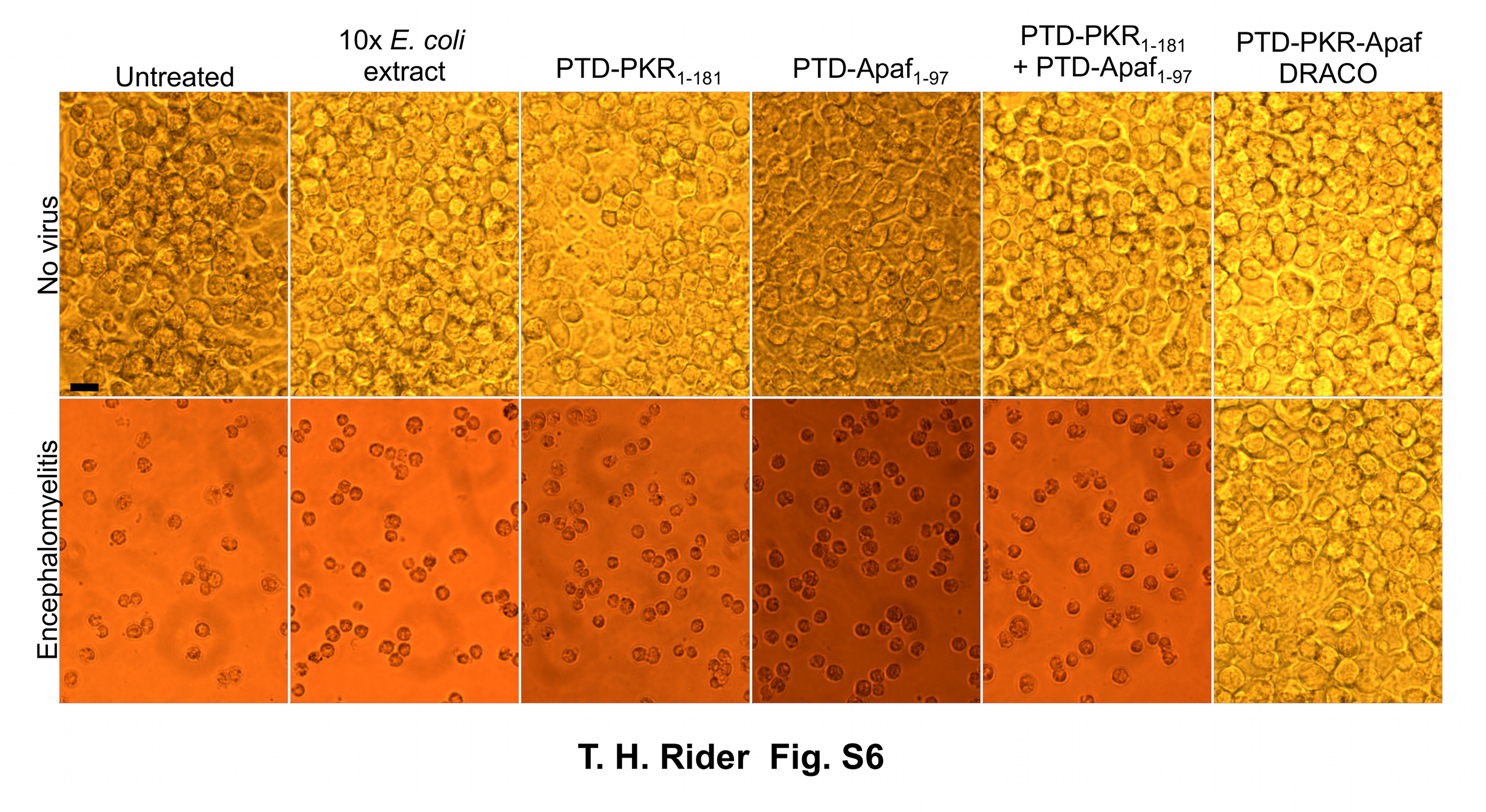

Supplement: Figure S6 — 200 nM PTD-PKR-Apaf DRACO was effective against murine encephalomyelitis in L929 cells. Representative photographs were taken 21 days after challenge with 50 pfu/well. Scale bar = 25 µm. (TIF) [file pone.0022572.s006.tif]

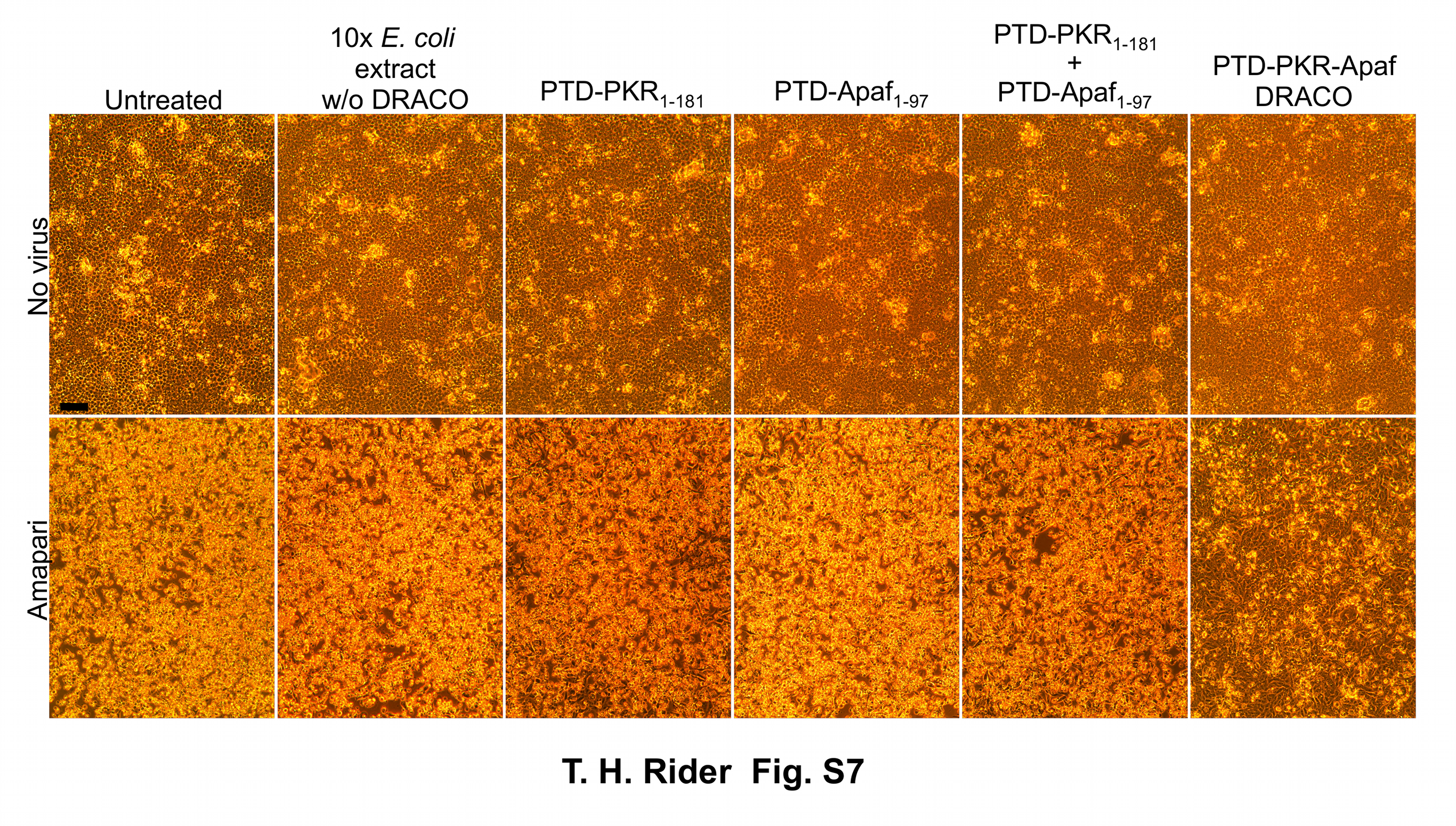

Supplement: Figure S7 — 100 nM PTD-PKR-Apaf DRACO was effective against Amapari arenavirus in Vero E6 cells. Representative photographs were taken 11 days after challenge with 300 pfu/well. Scale bar = 100 µm. (TIF) [file pone.0022572.s007.tif]

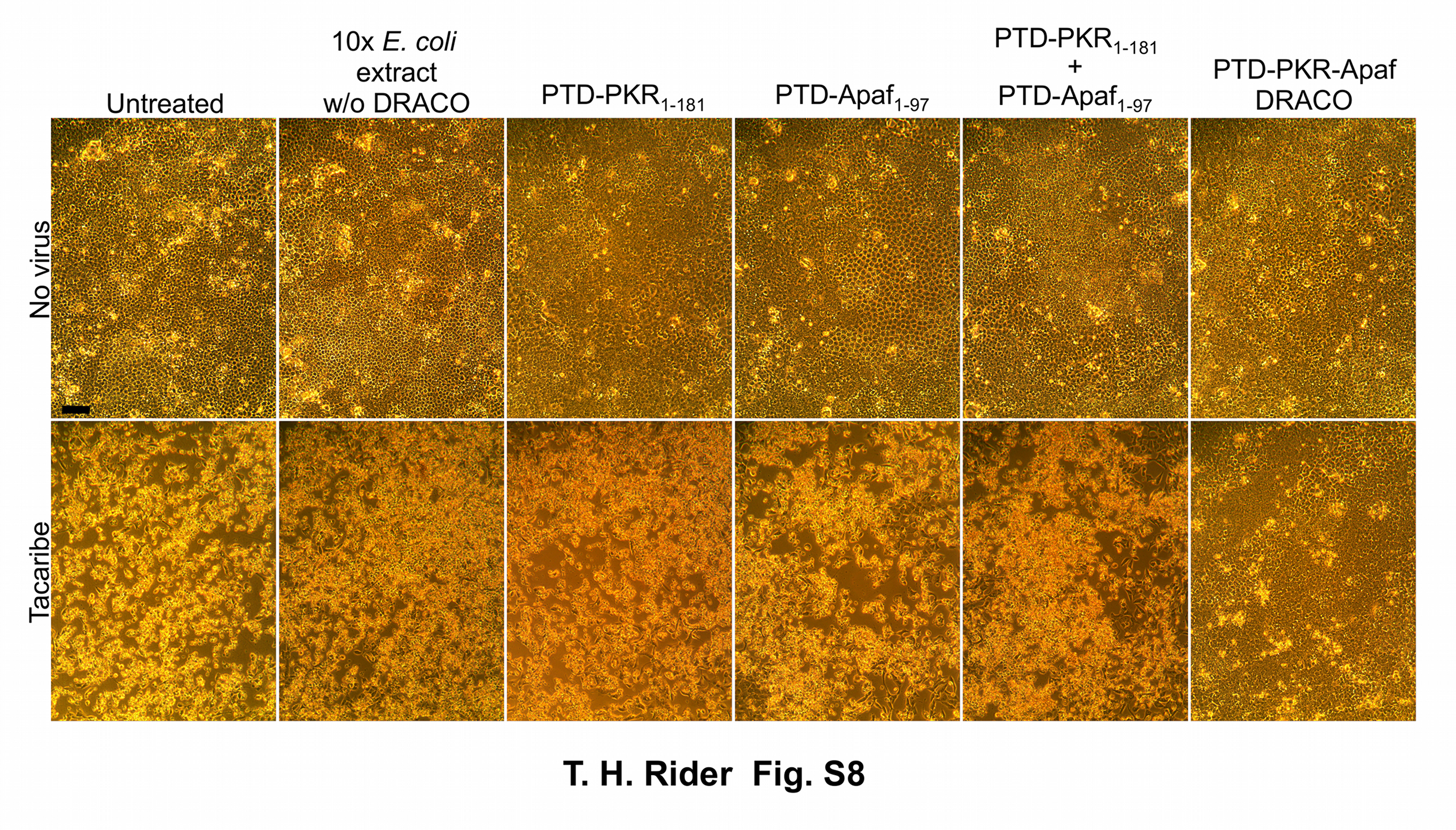

Supplement: Figure S8 — 100 nM PTD-PKR-Apaf DRACO was effective against Tacaribe arenavirus in Vero E6 cells. Photographs were taken 8 days after challenge with 140 pfu/well. Scale bar = 100 µm. (TIF) [file pone.0022572.s008.tif]

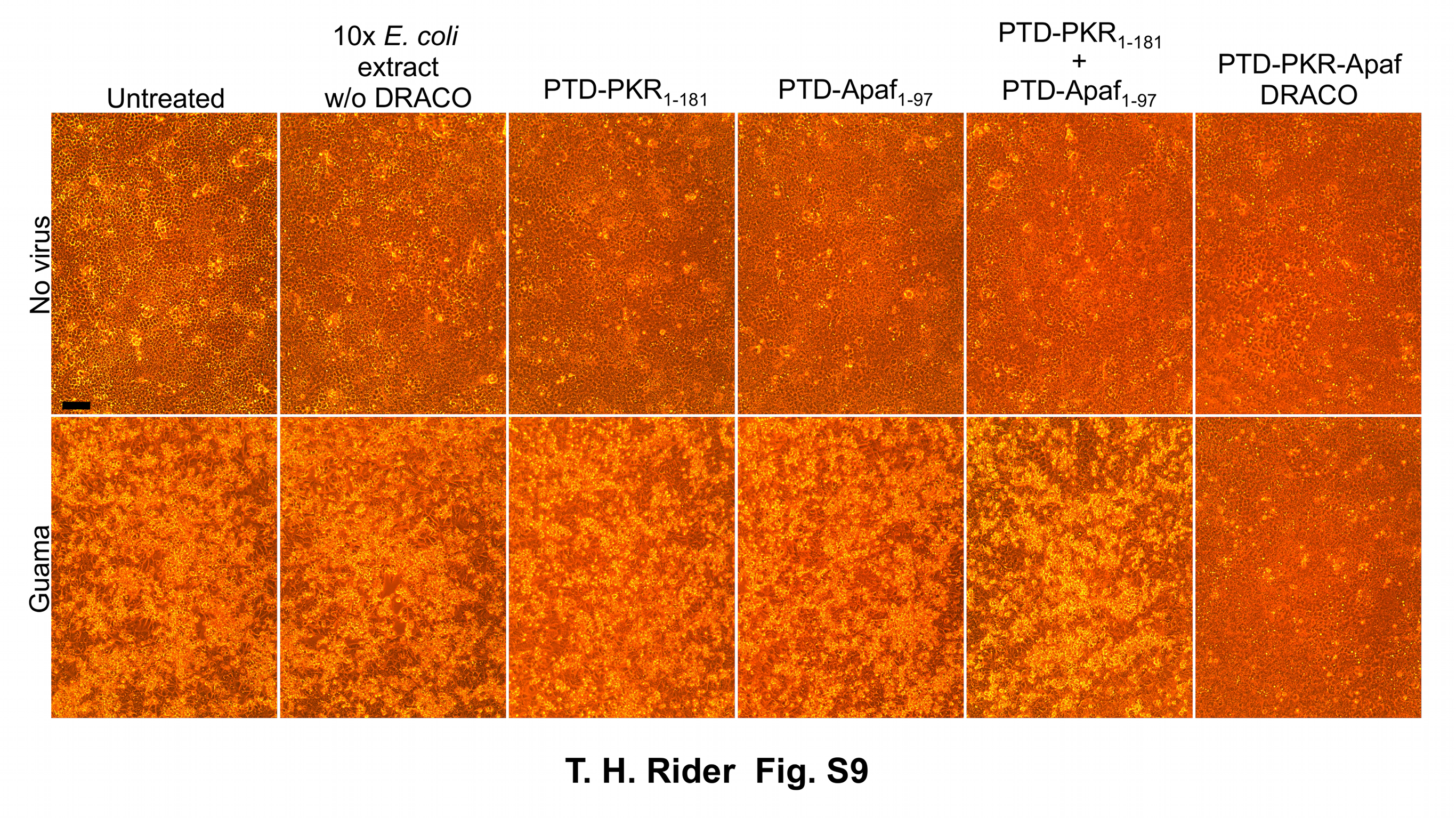

Supplement: Figure S9 — 200 nM PTD-PKR-Apaf DRACO was effective against Guama Be An 277 bunyavirus in Vero E6 cells. Photographs were taken 4 days after challenge with 30 pfu/well. Scale bar = 100 µm. (TIF) [file pone.0022572.s009.tif]

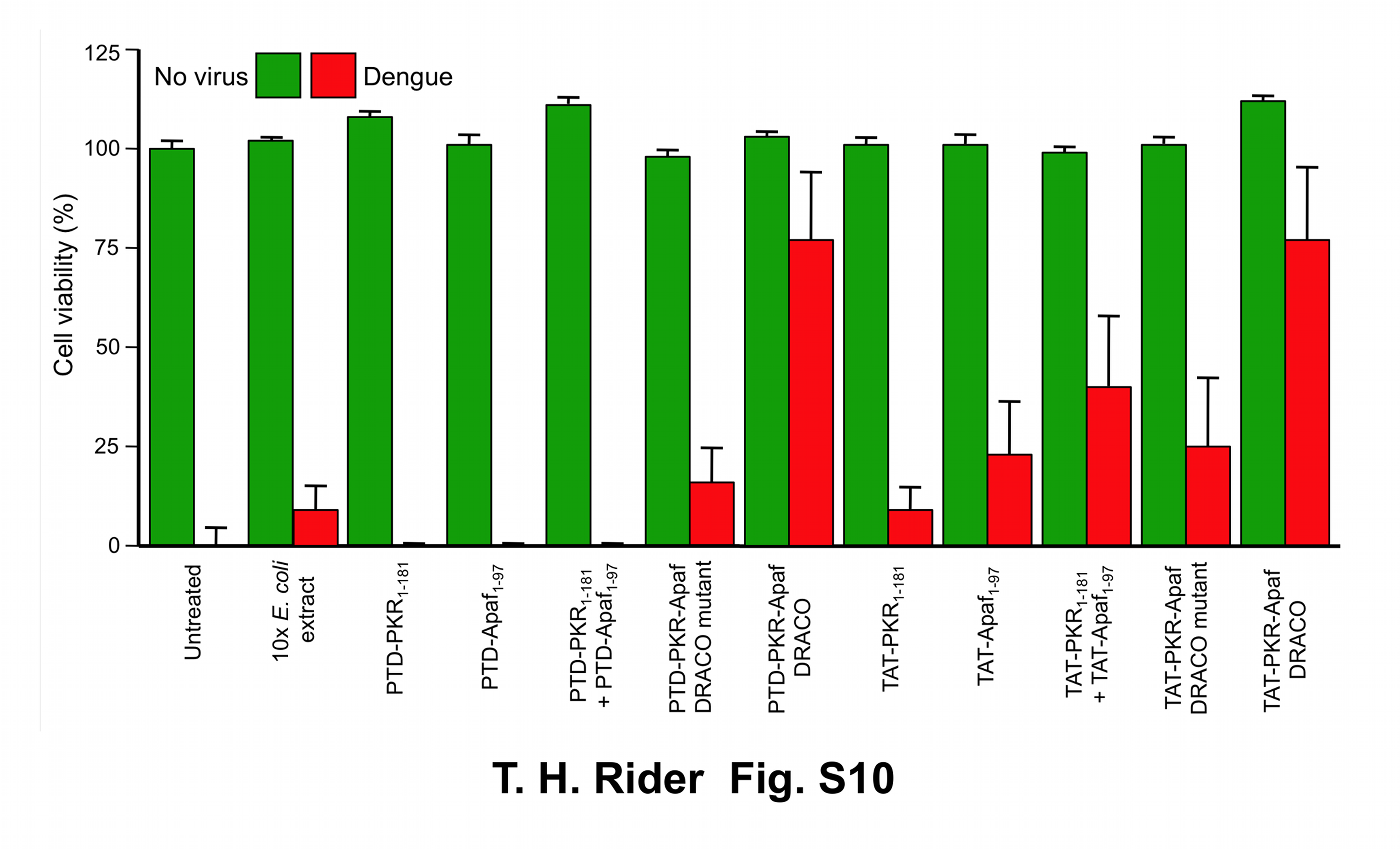

Supplement: Figure S10 — 200 nM PKR-Apaf DRACO was effective against dengue flavivirus in Vero E6 cells. Cell viability was measured 18 days after challenge with 16 pfu/well. (TIF) [file pone.0022572.s010.tif]

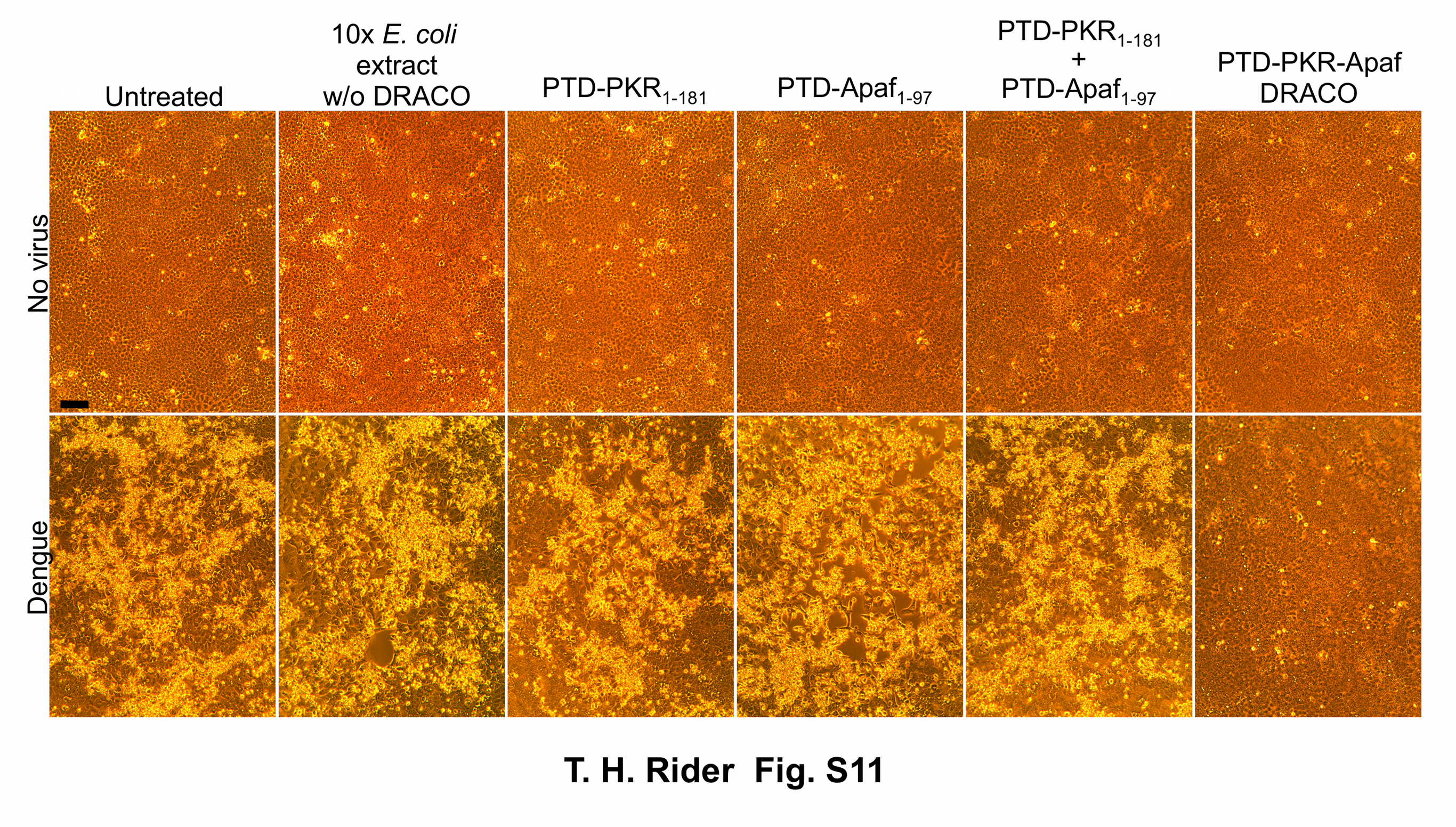

Supplement: Figure S11 — 100 nM PTD-PKR-Apaf DRACO was effective against dengue flavivirus in Vero E6 cells. Photographs were taken 7 days after challenge with 160 pfu/well. Scale bar = 100 µm. (TIF) [file pone.0022572.s011.tif]

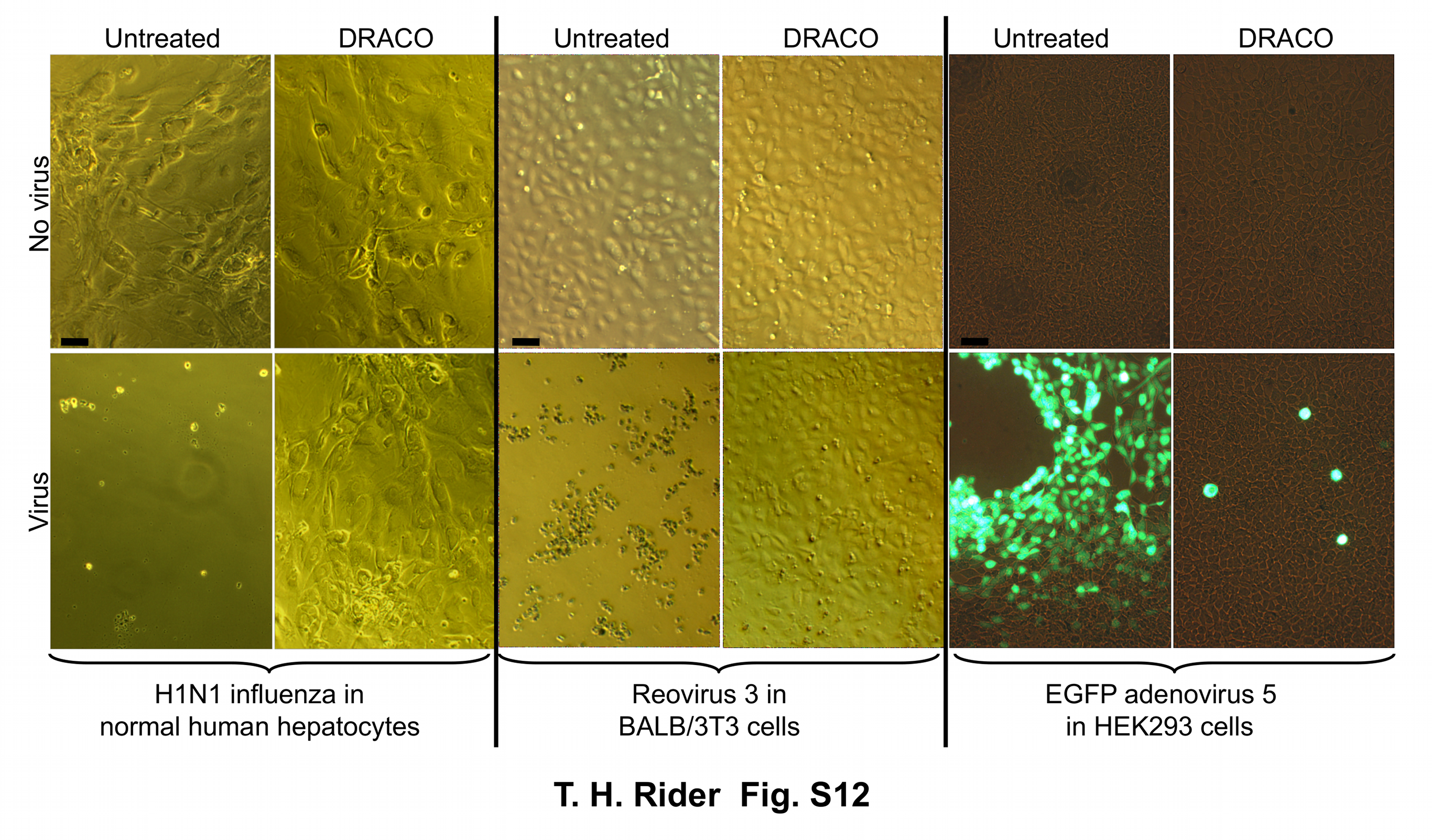

Supplement: Figure S12 — DRACOs were effective against a broad spectrum of other viruses in a variety of cell types. Left four photos: 100 nM PKR-Apaf DRACO was effective against H1N1 influenza A/PR/8/34 in normal human hepatocytes. Untreated cells challenged with 105 pfu/well died within 3 days, whereas treated challenged cells were cultured for 72 days with no sign of viral CPE. Center four photos: 100 nM PTD-PKR-Apaf DRACO was effective against reovirus 3 in BALB/3T3 murine cells. Photographs were taken 11 days after challenge with 30 pfu/well reovirus 3. Right four photos: 200 nM PTD-2×E3L-Apaf DRACO was effective against adenovirus 5 in human embryonic kidney AD293 cells. Fluorescent microscope photographs were taken 4 days after challenge with 25 pfu/well adenovirus 5 expressing enhanced green fluorescent protein (EGFP). Scale bars = 50 µm. (TIF) [file pone.0022572.s012.tif]
